# RNA quantification using gold nanoprobes - application to cancer diagnostics

**DOI:** 10.1186/1477-3155-8-5

**Published:** 2010-02-24

**Authors:** João Conde, Jesús M de la Fuente, Pedro V Baptista

**Affiliations:** 1 CIGMH, Departamento de Ciências da Vida, Faculdade de Ciências e Tecnologia, Universidade Nova de Lisboa, Campus de Caparica, 2829-516 Caparica, Portugal; 2 Instituto de Nanociencia de Aragón, Universidad de Zaragoza, Pedro Cerbuna 12, 50009, Zaragoza, Spain

## Abstract

Molecular nanodiagnostics applied to cancer may provide rapid and sensitive detection of cancer related molecular alterations, which would enable early detection even when those alterations occur only in a small percentage of cells. The use of gold nanoparticles derivatized with thiol modified oligonucleotides (Au-nanoprobes) for the detection of specific nucleic acid targets has been gaining momentum as an alternative to more traditional methodologies. Here, we present an Au-nanoparticles based approach for the molecular recognition and quantification of the *BCR-ABL *fusion transcript (mRNA), which is responsible for chronic myeloid leukemia (CML), and to the best of our knowledge it is the first time quantification of a specific mRNA directly in cancer cells is reported. This inexpensive and very easy to perform Au-nanoprobe based method allows quantification of unamplified total human RNA and specific detection of the oncogene transcript. The sensitivity settled by the Au-nanoprobes allows differential gene expression from 10 ng/μl of total RNA and takes less than 30 min to complete after total RNA extraction, minimizing RNA degradation. Also, at later stages, accumulation of malignant mutations may lead to resistance to chemotherapy and consequently poor outcome. Such a method, allowing for fast and direct detection and quantification of the chimeric *BCR-ABL *mRNA, could speed up diagnostics and, if appropriate, revision of therapy. This assay may constitute a promising tool in early diagnosis of CML and could easily be extended to further target genes with proven involvement in cancer development.

## Background

The National Cancer Institute envisions that over the next years, nanotechnology will result in significant advances in early detection, molecular imaging, targeted and multifunctional therapeutics, prevention and control of cancer [1]. Nanodiagnostics is a burgeoning field as more and improved techniques are becoming available for clinical diagnostics with increased sensitivity at lower costs [2-10]. Due to their optical properties, gold nanoparticles (AuNPs) have been used for DNA/RNA screening approaches, namely via functionalization with thiolated oligonucleotides (Au-nanoprobes), capable of specifically hybridizing with a complementary oligonucleotide sequence [9].

**Figure 1:**
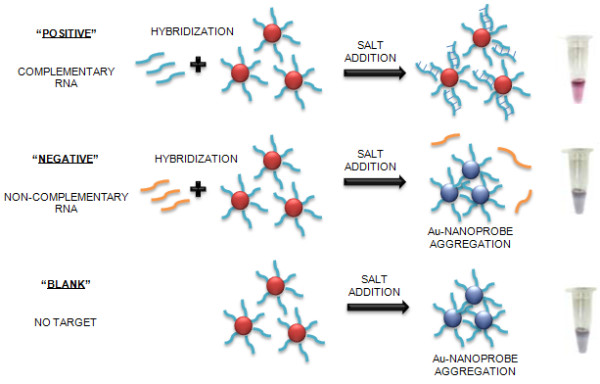
**Schematic representation of Au-nanoprobe assay method**. The assay is based on the increased stability of the Au-nanoprobes upon hybridization with the complementary RNA target in solution, while non-hybridized Au-nanoprobes easily aggregate once the solution's ionic strength is increased. Positive: sample in the presence of complementary RNA; Negative: sample in the presence of non-complementary RNA; Blank: Au-nanoprobe alone (no target).

The surface plasmon resonance (SPR) of AuNPs is responsible for the intense colors - monodisperse Au-nanoprobes (≈ 13 nm) appear red and exhibit a narrow SPR band centered around 520 nm; a solution containing aggregated Au-nanoprobes appears blue, due to a red shift of the SPR. Our method relies on visual and/or spectroscopy comparison of solutions before and after salt induced Au-nanoprobe aggregation -presence of complementary target prevents aggregation and the solution remains red (SPR peak at ± 520 nm); non-complementary targets do not prevent Au-nanoprobe aggregation, resulting in a visible change of color from red to blue (red-shift of the SPR peak to 600-650 nm) [5-7]. The principle of gold nanoparticles assay method detection of RNA hybridization is depicted in Figure 1. This non-cross-linking method has already been successfully applied for detection of eukaryotic gene expression without reverse transcription or PCR amplification steps [6], and for *Mycobacterium tuberculosis *detection [7,8].

Chronic myeloid leukemia (CML) is a clonal neoplastic disease of the hematopoietic stem cell, whose hallmark molecular event is the genetic t(9;22)(q34;q11) translocation known as the Philadelphia (Ph) chromosome [11,12]. This translocation - *ABL *gene (chromosome 9) and *BCR *gene (chromosome 22) - originates a *BCR-ABL *fusion gene, leading to the expression of a chimeric BCR-ABL protein with tyrosine-kinase activity [13-15]. The most commonly used procedures for the initial diagnosis and management of CML patients are expensive and time consuming, e.g karyotype analysis, reverse transcriptase polymerase chain reaction analyses (RT-PCR) and fluorescence in-situ hybridization (FISH) [16-18]. Therefore, there is a need for molecular methods able to detect and quantify the *BCR-ABL *fusion transcripts, which is of paramount relevance when monitoring minimal residual disease and genetic recurrence in patients known to harbor the translocation [19,20].

Here we present an Au-nanoprobe based approach for the molecular recognition and quantification of *BCR-ABL *b3a2 (e14a2) fusion for the early diagnosis of CML, which is inexpensive very easy to perform and uses total human RNA as target without reverse transcription and/or amplification.

## Methods

### Probe design and Au-nanoprobe synthesis

**Figure 2:**
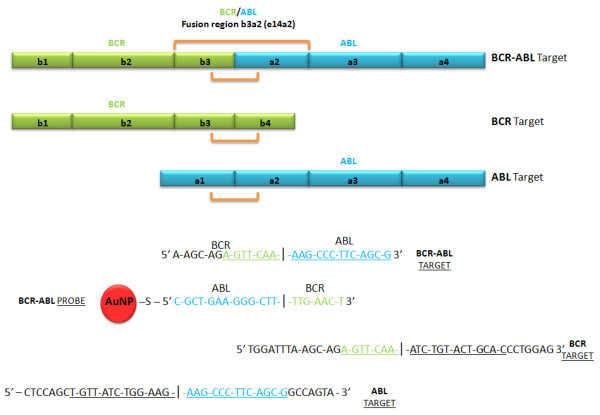
**Oligonucleotide probe and target sequences designed for *BCR-ABL *b3a2 (e14a2) junction and for *BCR *and *ABL *genes**. Complementary and non-complementary target sequences were used to study the level of specific interaction between the target and the Au-nanoprobes. *BCR-ABL *fusion positive (100% complementary); *BCR *and *ABL *gene sequences were used as controls (50% non-complementary); and a completely unrelated sequence (100% non-complementary) was used as negative control.

The probe sequence 5'-thiol-CGCTGAAGGGCTTTTGAACT-3' and the complementary target derive from the *BCR-ABL *b3a2 (e14a2) chimeric protein mRNA (Gene-Bank accession no. AJ 131466.1: 5'-TGGATTTAAGCAGAGTTCAAAAGCCCTTCAGCGGCCAGTA-3'), and the control oligonucleotide target sequences: *BCR *(Gene-Bank accession no. NM 021574.2: 5'-TGGATTTAAGCAGAGTTCAAATCTGTACTGCACCCTGGAG-3'), *ABL *(Gene-Bank accession no. NM 005157.3: 5'-CTCCAGCTGTTATCTGGAAGAAGCCCTTCAGCGGCCAGTA-3') and an unrelated target (5'-AGGAAAACGATTCCTTCTAACAGAAATGTCCTGAGCAATC-3'). The way these sequences relate to each other is illustrated in Figure 2.

The 13 nm gold nanoparticles were prepared by the citrate reduction method described by Lee and Meisel [21]. The thiolated oligonucleotide was dissolved in 1 ml of 0.1 M DTT, extracted three times with ethyl acetate, and further purified through a desalting NAP-5 column (Pharmacia Biotech, Sweden) according to the manufacturer's instructions. The Au-nanoprobe was prepared as described in Baptista et al [5]. Briefly, 500 μl of 10 μM thiol modified oligonucleotide was initially incubated with 6 ml of an aqueous solution of AuNPs (≈8.55 nM) for at least 16 h. After centrifugation (20 min at 14500 G), the oily precipitate was washed with 5 ml of 10 mM phosphate buffer (pH 8.0), 0.1 M NaCl, recentrifuged and redispersed in 5 ml of the same buffer to a final concentration in AuNPs of 8.5 nM. The resulting Au-nanoprobe was stored in the dark at 4°C.

### Cell culture and total RNA isolation

K562 erythroleukemic cells (*BCR-ABL *positive cell line derived from CML patients in blast crisis) and HL-60 cell line, a human leukemic promyelocytic cell line (*BCR-ABL *negative) were cultured in 90% RPMI 1640 and 10% FBS at 37°C with 5% CO_2_. *Saccharomyces cerevisae *cells were grown in YPD medium at 30°C overnight. Human peripheral blood mononuclear cells (PBMC) from control individuals were separated from 3 ml of heparinized peripheral venous blood by Ficoll gradient (Histopaque^®^-1077, Sigma-Aldrich, St. Louis, USA) according to manufacturer's specifications. Isolation of total RNA was performed using a High Pure RNA Isolation Kit (Roche Applied Science) according to the manufacturer's protocol. RNA concentration was determined by UV photometry and the RNA was stored at -80°C until use. RNA integrity was evaluated on a 1% agarose gel stained by GelRed™.

### Reverse transcription (RT) and PCR amplification

Total RNA extracted from K562 cells was subjected to RT with Revert-AidTM M-MuLV Reverse Transcriptase (Fermentas, Vilnius, Lithuania) according to the manufacturer's specifications, using 20 μM of BCR-ABLreverse primer, annealing at 42°C for 1 h and 70°C for 10 min to inactivate the reverse transcriptase. The reverse transcription reaction product, a 273-bp fragment of the human *BCR-ABL *fusion gene (b3a2 junction), was PCR amplified using primers BCR-ABLforward (18 nt): 5'-AGTCTCCGGGGCTCTATG-3' and BCR-ABLreverse (20 nt): 5'-GATTATAGCCTAAGACCCGG-3'. PCR amplification of the b3a2 region was carried out in 25 μl using 0.25 μM of primers, 0.2 mM dNTPs with 1 U Taq DNA polymerase (Amersham Biosciences, GE Healthcare, Europe, GmbH). The PCR reactions were performed in duplicate on a MyCycler Thermocycler (Bio-rad). Thermal cycling conditions consisted of denaturation at 95°C for 5 min and 30 cycles of amplification, each cycle consisting of denaturation of 95°C for 30 s, annealing at 52°C for 30 s, elongation was at 72°C for 30 s and final elongation at 72°C for 5 min and cooling at 4°C. The sequence of the PCR products was confirmed by sequencing.

### Real-Time RT-PCR assay

The Real-Time PCR amplification was performed in a Corbett Research Rotor-Gene RG3000 using SYBR GreenER Real-Time PCR Kit (Invitrogen, Karlsbad, CA, USA) according to manufacturer's specifications in 50 μl reactions containing cDNA from K562 and HL-60 cell-lines, 1× SYBR Green SuperMix and 200 nM of BCR-ABLforward and BCR-ABLreverse. The amplification conditions consisted of 50°C for 2 min hold, 95°C during 10 min hold, followed by 40 cycles consisting of denaturation at 95°C for 30 s, annealing at 52°C for 30 s, extension at 72°C for 30 s, with a final extension step at 72°C for 5 min. All the results were originated from three independent experiments.

### Au-nanoprobe hybridization and color detection

The Au-nanoprobe assay was performed in a total volume of 30 μl containing the Au-nanoprobe at a final concentration of 2.5 nM, the appropriate targets at a final concentration of 100 fmol/μl (100% complementary *BCR-ABL *target; 50% complementary *BCR *and *ABL *targets, and 100% non-complementary target) in 10 mM phosphate buffer (pH 8.0). Total RNA was used at a final concentration 10-60 ng/μl [100% complementary K562 cells RNA (*BCR-ABL *Positive); non-complementary HL-60 cells RNA (*BCR-ABL *Negative)]. Blank measurements were made in exactly the same conditions but replacing target or total RNA for an equivalent volume of 10 mM phosphate buffer (pH 8.0).

Following 5 min of denaturation at 95°C, the mixtures were allowed to stand for 30 min at 25°C and 0.3 M MgCl_2 _was added at a final concentration of 0.16 M. After 15 min at room temperature for color development, photographs were taken and assayed by UV-visible spectroscopic measurements of the SPR band. Absorption spectra were performed in a UNICAM, model UV2, UV-visible spectrophotometer with Ultra-Micro quartz cells (Hellma, Germany), using 10 mM phosphate buffer (pH 8.0), 0.1 M NaCl as reference. The areas under the curve (AUC_500 nm-560 nm_/AUC_570 nm-630 nm_) were calculated with the values for absorbance for 500 nm-600 nm/570 nm-630 nm using the trapezoidal rule.

## Results and Discussion

### Gold nanoprobe assay for target detection

**Figure 3:**
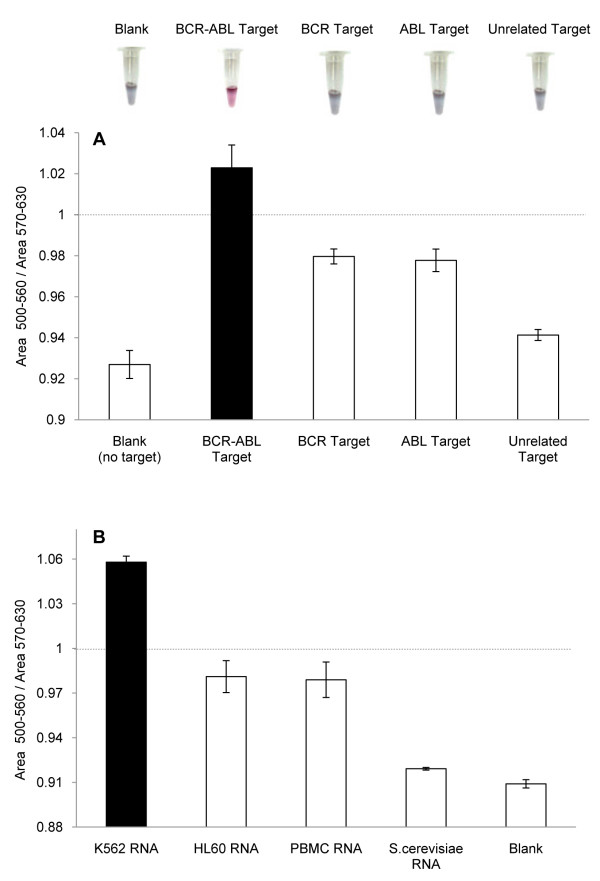
**Au-nanoprobe detection of the *BCR-ABL *fusion gene sequence**. (**A**) Colorimetric assay (above) and respective spectrophotometry (below) relative to the detection of synthetic *BCR-ABL *oligonucleotide target. Oligonucleotides with *BCR *or *ABL *sequence only (showing 50% complementarity) were used as controls and an unrelated target (showing 100% non-complementarity to the Au-nanoprobe) as negative control. (**B**) Detection of *BCR-ABL *in total RNA from K562 cell line, HL-60 cell line and human PBMC (harboring 50% complementary targets to the nanoprobe) and *S. cerevisiae *cells (100% non-complementary). Nanoprobe aggregation as measured by ratio of AUC_500 nm-560 nm_/AUC_570 nm-630 nm_. The dashed line represents the threshold of 1 considered for discrimination between Positive and Negative. The error bars represent the standard deviation from three independent assays.

First, we used thiolated ssDNA, complementary to the fusion site of the *BCR-ABL *mRNA, to functionalize gold nanoparticles and produce specific Au-nanoprobes. These nanoprobes were assessed in terms of specificity by means of total RNA mixtures spiked in with synthetic oligonucleotides harboring the fusion site *BCR-ABL *b3a2. It should be noted that, in reality, patients may only harbor one copy of the fusion gene and the remaining copies of normal *ABL *and *BCR *should be still functional, thus expressing the normal mRNA sequence. Two oligonucleotides, each harboring the normal sequence of the *BCR *and *ABL *genes respectively, were used to evaluate the probe's capability to discriminate from similar sequences. Following salt addition, the presence of the respective complementary synthesized target, protected the Au-nanoprobes from aggregation and the solution remained red; whereas the presence of non-complementary targets does not protect from aggregation and the solution turned blue (*BCR *and *ABL *controls only 50% complementary to the Au-nanoprobe) - Figure 3A. Absence of any target results in extensive aggregation (Blank). Only full hybridization of the Au-nanoprobe to a fully complementary synthetic sequence (*BCR-ABL *fusion sequence) avoids aggregation, whereas semi-complementary targets (normal *ABL *and *BCR *gene sequence) do not show the same capability.

**Figure 4:**
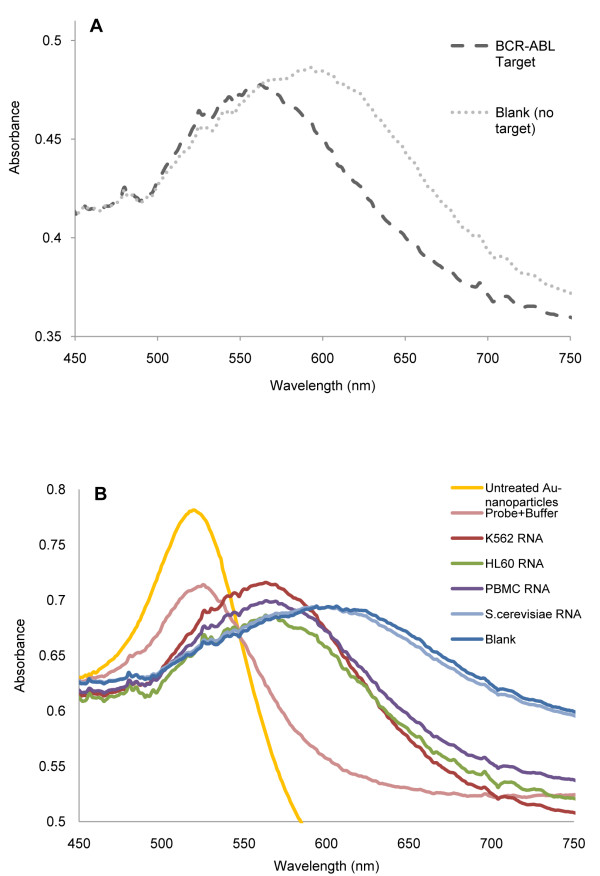
**Au-nanoprobe UV/Vis spectra obtained after inducing aggregation**. (**A**) UV/Vis spectra in absence (Blank) and in presence of target (*BCR-ABL *target). (**B**) UV/Vis spectra for the detection of the *BCR-ABL *b3a2 fusion mRNA in total RNA from K562 cells (*BCR-ABL *positive cell line), HL-60 cells (*BCR-ABL *negative cell line), human PBMC and *S. cerevisiae *cells; Au-nanoprobe alone before (Au-nanoprobe + buffer) and after (Blank) salt addition. All samples in 10 mM phosphate buffer (pH 8.0). Also, spectral data from untreated Au-nanoparticles in sodium citrate.

Based on the UV/Vis spectra (see Figure 4) obtained after inducing aggregation, Au-nanoprobe aggregation was evaluated in terms of SPR variation, i.e. a ratio between the free and aggregated fractions after 15 min incubation with [MgCl_2_] = 0.16 M. The ratio between the areas under the curve of the SPR was calculated using the trapezoidal rule - AUC_500 nm-560 nm_/AUC_570 nm-630 nm_. A ratio of 1 may be considered as the point of equilibrium between non-aggregated and aggregated nanoprobe, hence the threshold to respectively consider the positive and negative discrimination of sequences (positive identification of complementary target ratio >1). Commonly, for discriminating between two significantly different aggregation levels, as for example in a YES/NO for identification of a given target, the ratio between the peaks at 520 nm and 600 nm is usually used. However, for identifying small differences in aggregation levels between two quantities for the same target, there is a need to decrease the noise level in the spectra. When establishing a ratio between two absorbance values, the error increases mainly due the noise in the spectra, which can be overcome (i.e. strongly reduced) by using an integral of the signal, i.e. the area under the curve.

The Au-nanoprobes were then used for the detection of the *BCR-ABL *b3a2 fusion mRNA in total RNA extracted from K562 cells (*BCR-ABL *positive cell line), HL-60 cells (*BCR-ABL *negative cell line), human peripheral blood mononuclear cells (PBMC) and *S. cerevisiae *cells - Figure 3B. Total RNA from HL-60 cell line and PBMC only express the normal *BCR *and *ABL *transcripts, which are 50% complementary to the probe sequence. Total RNA from an unrelated organism (*S. cerevisae*) was used to confirm specificity of the detection method. The results originate from a minimum of three individual parallel hybridization experiments. *BCR-ABL *fusion discrimination was observed only for samples containing the complementary RNA target (K562 cells). Samples containing the normal *BCR *and *ABL *genes showed a minor stabilization of the Au-nanoprobe, yet below the threshold for positive identification of the target (ratio <1).

### Gold nanoprobe assay for RNA quantification

**Figure 5:**
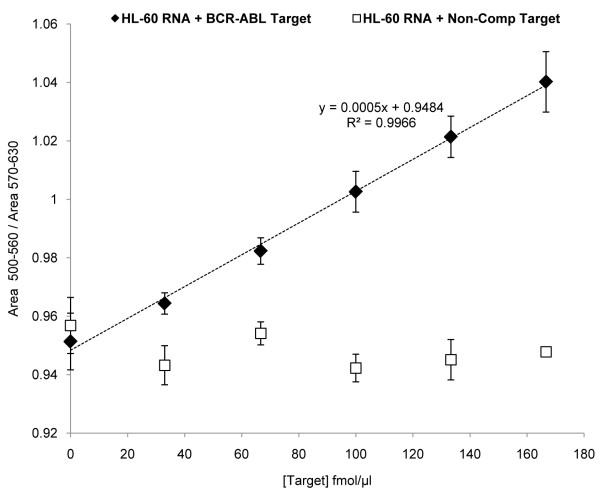
**Quantification of *BCR-ABL *by Au-nanoprobe**. Ratio AUC_500 nm-560 nm_/AUC_570 nm-630 nm _as function of specific target concentration in mixtures of 20 ng/μl total RNA from *BCR-ABL *negative cell line HL-60 spiked in with increasing concentrations of the synthetic oligonucleotide (black diamond's - complementary target; blank squares - non-complementary target). The error bars represent the standard deviation from three independent assays.

Once the specific identification of the target sequence was achieved, the Au-nanoprobes were used to evaluate both the limit of detection and quantification potential. For this purpose, different concentrations of the specific synthetic oligonucleotide target were used to spike in 20 ng/μl of total RNA extracted from the *BCR-ABL *negative cell line HL-60. Our data indicate a linear correlation (R^2 ^= 0.9966) between the AUC_500 nm-560 nm_/AUC_570 nm-630 nm _for target concentration range between 33 and 133 fmol/μl (Figure 5). A non-complementary target was used in a parallel spike in experiment, where extensive aggregation of the Au-nanoprobe was observed for all tested concentrations.

**Figure 6:**
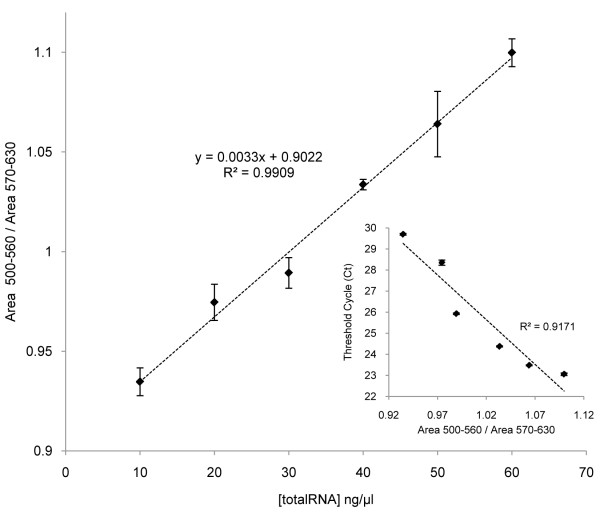
**Au-nanoprobe based quantification of *BRC-ABL *fusion mRNA directly in total RNA extracted from K562 cell line**. Nanoprobe aggregation as measured by ratio of AUC_500 nm-560 nm_/AUC_570 nm-630 nm _for increasing concentrations of total RNA from a *BCR-ABL *positive cell line (K562) - 10 to 60 ng/μl. (**Inset**) Real-Time RT-PCR *vs. *Au-Nanoprobe Assays. A linear association (R^2 ^= 0.9171) was found between the two methods. The error bars represent the standard deviation from three independent assays.

In order to validate the detection and quantification potential of the Au-nanoprobes in the positive cell line (K562), Real-time RT-PCR was used. Our method showed a linear correlation for *BCR-ABL *detection within the range of 10-60 ng/μl of total RNA (see Figure 6). A linear association (R^2 ^= 0.9171) was found between the two methods, Real-Time RT-PCR and Au-nanoprobe, for *BCR-ABL *detection (**inset in Figure **6). Real-Time RT-PCR is a more robust and sensitive technique but time consuming, more expensive and requiring expensive equipment and highly trained personnel.

## Conclusions

In conclusion, we demonstrated the potential of an Au-nanoprobe based assay for the specific identification and quantification of aberrant expression of genes involved in cancer development. This Au-nanoprobe strategy allowed for detection of less than 100 fmol/μl of a specific RNA target, with the possibility of discriminating between a positive and negative from as little as 10 ng/μl of total RNA. As proof-of-concept we used the *BCR-ABL *fusion product that is of paramount importance in chronic myeloid leukemia, showing the application potential in cancer diagnosis. To our knowledge, this is the first report on quantification of human mRNA directly from total RNA without reverse transcription or amplification. The assay has a total work-up time of less than 45 minutes with comparable sensitivity to those demonstrated by traditional molecular biology methodologies.

## List of Abbreviations

(CML): Chronic myeloid leukemia; (AuNPs): Gold nanoparticles; (Au-nanoprobes): Gold nanoprobes; (SPR): Surface plasmon resonance; (Ph) chromosome: Philadelphia; (PBMC): Peripheral blood mononuclear cells; (AUC): Area under the curve.

## Competing interests

The authors declare that they have no competing interests.

## Authors' contributions

JC participated in the sequence alignment and design of the nanoprobe, carried out the nanoprobe synthesis, and performed the detection assays. JF participated in the design of the study. PB conceived the study, participated in its design and coordination, and drafted the manuscript. All authors read and approved the final manuscript.
